# Toll-like receptor-4 pathway is required for the pathogenesis of human chronic endometritis

**DOI:** 10.3892/etm.2014.1990

**Published:** 2014-09-24

**Authors:** JINFEN JU, LIANGPENG LI, JINGYAN XIE, YAN WU, XI WU, WEIHON LI

**Affiliations:** 1 Department of Gynecology, Nanjing Municipal Organ Hospital, Nanjing, Jiangsu 210018, P.R. China; 2 Department of Thoracic and Cardiovascular Surgery, Nanjing Hospital Affiliated to Nanjing Medical University, Nanjing, Jiangsu 210006, P.R. China; 3 Department of Gynecology and Obstetrics, Nanjing Hospital Affiliated to Nanjing Medical University, Nanjing, Jiangsu 210006, P.R. China

**Keywords:** Toll-like receptor 4, chronic endometritis, inflammation, signaling pathway

## Abstract

Toll-like receptor (TLR) signal transduction is a central component of the primary innate immune response to pathogenic challenge. TLR4, a member of the TLR family, is highly expressed in the endometrial cells of the uterus and could thus be a key link between human chronic endometritis (CE) and the immune system. However, the exact biological function of TLR4 in human CE remains largely unexplored. The present study aimed to examine the role of TLR4 in human CE. A comprehensive expression and activation analysis of TLR4 in the endometrial cells of the uterus from patients with human CE (n=25) and normal endometrial (NE) tissue (n=15) was performed. Western blot analyses demonstrated that compared with NE, the protein expression TLR4 markedly increased in human CE. Endometrial tissue scrapings were also used for total RNA extraction and were transcribed and amplified by reverse transcription quantitative polymerase chain reaction. The results showed that significant upregulation of tumor necrosis factor-α (TNF-α) and interleukin-1β (IL-1β), and downregulation of IL-10 mRNA was observed in CE compared with the NE group. Furthermore, the protein of the signaling adapter myeloid differentiation factor-88 and the accessory molecules (TNF receptor associated factor 6 and transforming growth factor-β-activated kinase 1) were also detected in all the assayed tissues. Of note, differential expression (CE versus NE) was observed by immunoblotting at each level of the nuclear factor-κB signaling cascade, including inhibitor κBα and P65 (all P<0.05). The altered TLR4 and its corresponding downstream signaling molecules in CE cells may be of relevance for the progression of the human CE. These findings indicate that the evaluation of expression patterns of TLR4 holds promise for the treatment of human CE.

## Introduction

Microbial disease of the female genital tract is common and of significant economic importance in humans. Microbial infections of the genital tract often infect the endometrium of humans to cause endometritis, uterine disease and infertility (1,2). Under normal pathophysiological conditions, a number of the mechanisms underlying the recognition of microbial pathogens by the innate immune system in vertebrates have been identified during the past 15 years (3,4). These mechanisms of innate immunity are not only important for classic immune cells, including neutrophils and macrophages, but are also evident in the endometrial and ovarian cells of mammals. At the cellular level, human chronic endometritis (CE) is characterized by inflammation with the elaboration of cytokines, chemokines and prostaglandins (2). Although the multiple signaling mechanisms that control the inflammatory response in the endometrium have been extensively studied, the molecular mechanisms that mediate the development of human CE are not completely understood. Thus, there is significant interest in identifying novel mechanisms for use in therapeutic interventions for treatment of human CE.

The initial immune defense of the endometrium against microbes is now considered to be highly dependent on pattern recognition receptors (5,6). Immune cells possess pattern recognition receptors, such as the Toll-like receptors 1–10 (TLR1-10) and several nucleotide-binding domain-like receptors (NLRs), which bind molecules specific to microbial organisms often known as pathogen-associated molecular patterns or microbial-associated molecular patterns (7). Briefly, TLR1, TLR2 and TLR6 recognize bacterial lipids, including lipoteichoic acid from Gram-positive bacteria and TLR5 binds flagellin. Nucleic acids, often from viruses, are recognized by TLR3, TLR7, TLR8 and TLR9, although TLR9 also recognizes bacterial DNA. The NLRs are also intracytoplasmic receptors that recognize bacterial peptidoglycans and components of viruses. Notably, TLR4, in complex with cluster of differentiation 14 and MD-2, binds lipopolysaccharide (LPS) on immune cells (8,9). Activation of pattern recognition receptors initiates the production of pro-inflammatory cytokines, chemokines and prostaglandins, often via the mitogen-activated protein kinase (MAPK) and nuclear factor-κB (NF-κB) pathways, leading to the recruitment of neutrophils and monocytes to the site of infection (7,9). Although the endometrial and systemic innate immune responses have been investigated in humans with endometritis, there are no studies associating TLR4 with induced infectious endometritis in human susceptible to persistent endometritis. The present study featured the following objectives: i) To determine whether TLR4 is altered in the endometrial cells of the uterus collected from human patients with CE and normal endometrial (NE) tissue; and ii) to explore the possible mechanisms that would be involved in any such effects that are observed.

## Materials and methods

### Study population and sample collection

Between January 2012 and December 2013, a total of 25 patients with CE underwent in-office diagnostic hysteroscopy and endometrial biopsy at the Nanjing Government Hospital (Nanjing, China). The diagnostic standard was as follows: Normal menstrual cycle, normal basis endocrine, no evident organ lesions found in the uterus and ovaries by ultrasound, no use of relevant sex hormone drugs for three months and tubal patency. A group comprising 15 patients with NE tissue was enrolled in parallel, and all the patients with CE and NE were included for further analyses. All the cases were diagnosed using clinical and pathological evidence. The study conformed to the principles outlined in the Declaration of Helsinki. All the retrospective reviews of the clinical data involving human samples were approved by the Human Research Ethics Committee of the Nanjing Government Hospital, and written informed consent was obtained from each patient.

### Reagents

Antibodies against the following proteins were purchased from Santa Cruz Biotechology, Inc (Santa Cruz, CA, USA): TLR4 (1:800), total-P65 (T-P65) (1:800), inhibitor (I)κBα (1:500), phosphorylated-P65 (P-P65) (1:300) and GAPDH (1:4000). The antibodies against myeloid differentiation factor-88 (MyD88) (1:1000), tumor necrosis factor (TNF) receptor-associated factor 6 (TRAF6) (1:800) and transforming growth factor-β-activated kinase 1 (TAK1) (1:800) were purchased from Cell Signaling Technology, Inc. (Danvers, MA, USA).

### Reverse transcription quantitative polymerase chain reaction (RT-qPCR)

For RT-qPCR (10), total RNA was extracted from the frozen human tissue using TRIzol^®^ (Invitrogen Life Technologies, Carlsbad, CA, USA) and reverse-transcribed into cDNA using oligo (dT) primers with a Transcriptor First Strand cDNA Synthesis kit. PCR amplifications were quantified using the SYBR Green PCR Master Mix (Applied Biosystems, Inc., Foster City, CA, USA) and normalized to *GAPDH* gene expression. The primers for the RT-qPCR are shown in Table I.

### Western blotting

Total proteins extracted from the left ventricle tissue and cultured cardiomyocytes were first lysed in radioimmunoprecipitation assay lysis buffer and the protein concentrations were measured using the Pierce^®^ Bicinchoninic Protein Assay kit (Pierce Biotechnology, Inc., Rockford, IL, USA). The protein extract (50 μg) was run on 8–12% SDS-PAGE gels (Invitrogen Life Technologies) and transferred to polyvinylidene difluoride membranes (Millipore, Billerica, MA, USA). The membranes were blocked in Tris-buffered saline with Tween 20 (TBST) containing 5% skimmed milk powder for 1 h at room temperature and incubated with various primary antibodies overnight at 4°C. Following incubation with secondary antibodies for 1 h at room temperature, membranes were washed with TBST four times, as previously described (11). Specific proteins were detected using an enhanced chemiluminescence reagent (GE Healthcare, Piscataway, NJ, USA) and captured on Hyperfilm (GE Healthcare). The results were analyzed through the Quantity One software (Bio-Rad, Hercules, CA, USA) for the semi-quantitation of the mean gray value of each blot. The specific protein expression levels were normalized to the GAPDH present on the same nitrocellulose membrane. All the presented results are representative of at least three independent experiments.

### Statistical analysis

The data are presented as the mean ± standard deviation. For two-group comparisons, Gaussian samples were compared using the two-tailed Student’s t-test, while non-Gaussian samples were compared using the non-parametric Mann-Whitney U test. Statistical analyses were performed using SPSS 17.0 software (SPSS, Inc., Chicago, IL, USA). P<0.05 was considered to indicate a statistically significant difference.

## Results

### TLR4 expression is increased in human CE

**Figure 1:**
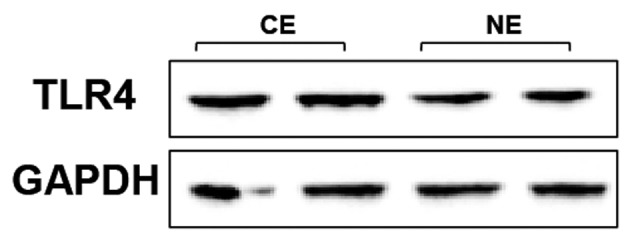
TLR4 expression is increased in human CE. Representative western blots from human CE and control NE tissues for the determination of the TLR4 protein levels expressed in the endometrial tissues (from three independent experiments). TLR4, Toll-like receptor-4; CE, chronic endometritis; NE, normal endometrial.

**Figure 2:**
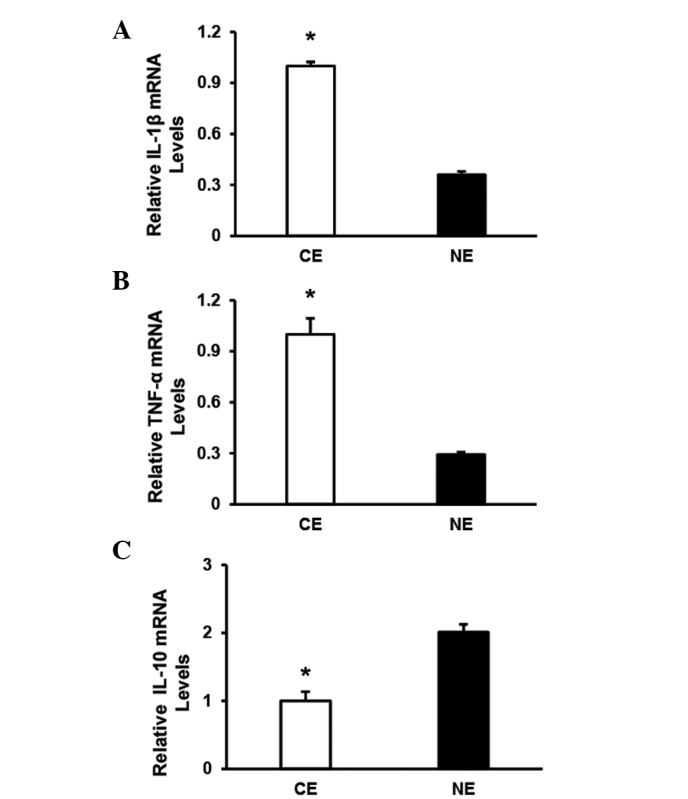
Expression of inflammatory markers in human CE. Reverse transcription quantitative polymerase chain reaction analysis of (A) *IL-1β*, (B) *TNF-α* and (C) *IL-10* in the indicated groups (n=4 per experimental group). Data represent the typical results of three or four different experiments, presented as the mean ± standard deviation. ^*^P<0.05 vs. the NE group. CE, chronic endometritis; NE, normal endometrial; *IL*, interleukin; *TNF-α*, tumor necrosis factor-α.

To investigate the potential role of TLR4 in the development of human CE, whether expression levels of TLR4 were altered in the pathological endometrium was first analyzed. The RT-qPCR and western blot analysis assays showed that the mRNA and protein expression levels of *TLR4* were significantly increased in human CE (n=7) compared with NE (n=5) (Fig. 1). In addition, the upregulation of *TLR4* in human CE was correlated with the induction of a series of inflammatory markers at the mRNA level, including interleukin (*IL*)-1β and *TNF-α* (Fig. 2A and B). However, the levels of *IL-10* were markedly decreased in human CE compared with NE (Fig. 2C). Collectively, the altered pattern of *TLR4* expression suggests that *TLR4* may be involved in the development of the inflammatory response in human CE.

### TLR4 regulates MyD88 signaling in human CE

**Figure 3:**
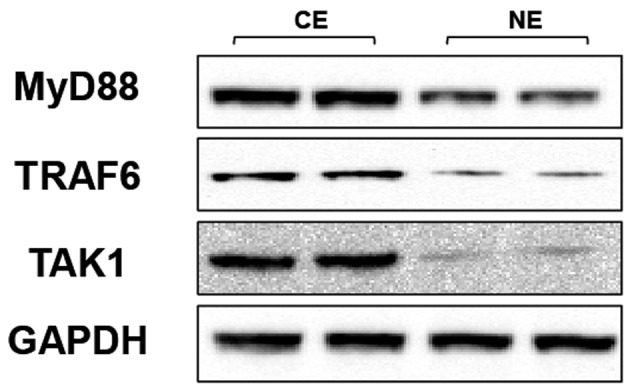
Toll-like receptor-4 regulates MyD88 signaling in human CE. Representative blots show the protein levels of MyD88, TRAF6 and TAK1 in endometrial tissues from human CE and control NE. MyD88, myeloid differentiation factor-88; TRAF6, tumor necrosis factor receptor-associated factor 6; TAK1, transforming growth factor-β-activated kinase 1; CE, chronic endometritis; NE, normal endometrial.

To dissect the possible molecular mechanisms through which the regulation of TLR4 affects the inflammatory response in human CE, the expressions/activities of TLR4 signaling molecules (the adapter protein MyD88 and the accessory molecules TRAF6 and TAK1) were investigated. The levels of MyD88, TRAF6 and TAK1 were clearer in human CE than NE, as shown by western blot analysis (Fig. 3). These results indicate that MyD88-TRAF6-TAK1 signaling is critical for the effect of TLR4 on human CE.

### TLR4-mediated inflammation is largely dependent on the promotion of NF-κB signaling in human CE

**Figure 4:**
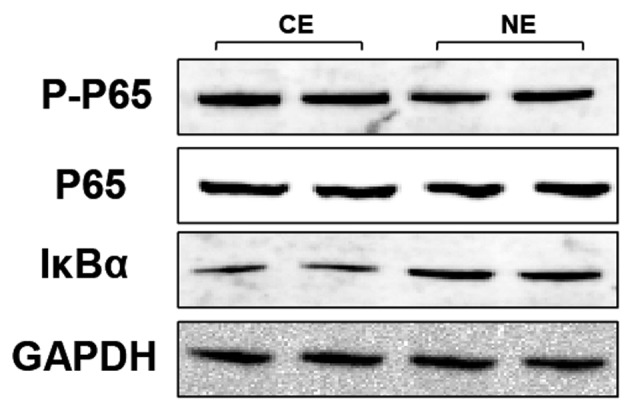
Toll-like receptor-4-mediated inflammation is largely dependent on the promotion of nuclear factor-κB signaling in human CE. Representative blots reveal the phosphorylated and total protein levels of P65 and IκBα in the human CE and NE groups. CE, chronic endometritis; NE, normal endometrial; P-, phosphorylated-; IκBα, inhibitor κBα.

Increasing evidence demonstrated that the NF-κB pathway is known to have an important role in inflammation, and the triggering of the TLR pathway leads to the activation of NF-κB and subsequent regulation of immune and inflammatory genes (12). The activation of the NF-κB pathway in the indicated groups was therefore investigated. By western blot analysis, expression levels of the T-P65, an important component that usually forms the most common heterodimers of NF-κB, was shown to not be different between the human CE and NE groups (Fig. 4). However, P-P65, comprising the functionally active form of the transcription factor in the nucleus, and IκBα, which binds with NF-κB to inhibit its activation, were also detected (Fig. 4). The results show that the expression of P-P65 (Fig. 4) was significantly higher in the human CE group compared with the NE group, while the expression pattern of IκBα was reversed among the two groups (Fig. 4). The protein expression of IκBα was highly increased in the human CE group compared with the NE group. Taken together, these data indicate that the expression levels of the NF-κB pathway is significantly correlated with TLR4 activation in human CE.

## Discussion

The results of the present study provide evidence of the critical role of TLR4 in human chronic endometritis. The level of TLR4 was observed to be significantly upregulated in human CE. In addition, it was found that the expression of MyD88, TRAF6 and TAK1 molecules are involved in the mechanism of TLR4 in human endometrial endothelial cell responses to bacterial infection. Furthermore, it was identified that TLR4 exerted a pro-inflammatory effect by the activation of NF-κB, thus facilitating its transcriptional activity. These results suggest that the TLR4-dependent NF-κB activation pathway contributes to the inflammatory response in human CE.

Bacterial infection of the female genital tract can cause pelvic inflammatory disease, infertility, endomyometritis, septic pelvic thrombophlebitis and even pregnancy complications, including preterm labor (8). Notably, the endometrium is a unique mucosa, which is normally sterile throughout pregnancy but becomes exposed to numerous bacteria during the postpartum period (13). Indeed, specific Gram-negative infections have been identified in the human endometrium (14). In previous years, increasing attention has been directed to innate immunity, which is the initial defense system against pathogens. In particular, TLRs, which comprise a family of membrane proteins consisting of ≥10 members, are indicated to play significant roles in innate immunity (9,15). The innate immune signaling also contributes to tissue homeostasis, including the microflora, proliferation and apoptosis of epithelial cells, and regeneration. However, abnormal activation of innate immune signaling may also cause sepsis, chronic inflammation, autoimmune diseases, tissue and organ injuries, fibrosis and carcinogenesis that are unfavorable to the host (16). Of note, a previous study by Krikun *et al* (17) demonstrated that *TLR4* mRNA and protein expression were reported in primary cultures of epithelial and stromal cells from the human endometrium. In addition, TLR4 can recognize chlamydial LPS and cHSP60 (18). The present study tested the hypothesis that TLR4-dependent signaling is essential for the response to infections by the epithelial cells of the human endometrium. TLR4 protein expression was detectable in all the tissue lines by western blotting, but the expression of TLR4 in the human CE endothelial cell was significantly higher than that of the NE. These data revealed that high TLR4 expression level are associated with human CE, which indicated the importance of TLR4 in inflammatory response progression. Notably, the TLR4 signaling pathway requires downstream adaptor molecules such as MyD88, that interact directly with the Toll-interleukin receptor domain of TLRs on the cell plasma membrane (19). Following recognition of ligands by TLRs, MyD88 recruits IL-1 receptor-associated kinase, which stimulates TRAF6, TAK1 and NF-κB-inducing kinase complex, leading to the activation of IκB kinases, which stimulate IκBα phosphorylation and degradation, resulting in NF-κB translocation to the nucleus, binding to target DNA sequences and stimulation of gene expression (20). In the present study, it was observed that MyD88 and the associated downstream molecules were also significantly augmented, suggesting that the MyD88-mediated signaling could be involved in stimulating an over-exuberant inflammatory response, possibly influencing disease progression.

To further understand the mechanism responsible for TLR4/MyD88 signaling, the activation of downstream NF-κB pathways was explored. NF-κB activation has been shown to play a critical role in regulating the expression of groups of genes involved in immune and inflammatory responses, cell death and survival, cell growth and the cell cycle. NF-κB is a critical transcription factor in TLR-mediated signaling pathways (21,22). The main pathway of TLR4-mediated signaling that leads to NF-κB activation involves the adaptor molecule termed MyD88 signaling complexes (23). In the present study, it was found that the expression levels of P-P65 was markedly upregulated in the human CE group compared with the control group. Unlike the other components of the NF-κB cascade, the decrease in expression of IκBα in the control group was reversed. This result could be explained by TLR4 inducing the NF-κB cascade through the phosphorylation and ubiquitination of IκBα to release NF-κB for its translocation to the nucleus. A previous study (24) confirmed that as there was more IκB-α degradation, the expression of the IκB-α protein would also decrease. Furthermore, consistent with the upstream NF-κB components, the assessment of the protein levels of the downstream pro-inflammatory cytokine markers (IL-1β and TNF-α) exhibited the same trends in the human endometrial tissue. By contrast, the anti-inflammatory cytokine IL-10 exhibited a significant decline. Based on the present results, it may be proposed that TLR4 has a pivotal role in the activities of the NF-κB signaling pathway, which in turn regulates the expression of genes involved in the inflammatory response in human CE.

In conclusion, the present study demonstrated that elevated TLR4 expression levels could be associated with the disease progression in patients with CE, which indicates that TLR4 may serve as a valuable marker in human CE. However, the possible underlying mechanisms for its participation in human CE progression is not entirely clear; therefore, other signaling pathways require investigating in order to gain an improved understanding of the molecular mechanism in this field.

## Figures and Tables

**Table I:** Primers for the reverse transcription quantitative polymerase chain reaction.

Name	Forward	Reverse
Interleukin-1β	CCGTGGACCTTCCAGGATGA	GGGAACGTCACACACCAGCA
Tumor necrosis factor-α	ACTGAACTTCGGGGTGATCGGT	TGGTTTGCTACGACGTGGGCTA
Interleukin-10	TGAATTCCCTGGGTGAGAAG	CTCTTCACCTGCTCCACTGC
*GAPDH*	ACTCCACTCACGGCAAATTC	TCTCCATGGTGGTGAAGACA
